# Pulmonary rehabilitation for post-TB lung disease led by TB survivors

**DOI:** 10.5588/pha.25.0001

**Published:** 2025-06-04

**Authors:** F.J. Mtei, I. Meadows, K. Msaji, F. Thobias, A. Liyoyo, A. Kimaro, P.M. Joseph, C. Gitige, O. Kaswaga, S. Matoi, A. Ngoma, A. Mbuya, P. Mbelele, L. Ritte, L. Subi, P. Neema, R. Kisonga, D. Mbwana, E. Mpolya, M. Drage, L.I. Lochting, S.K. Heysell, S.G. Mpagama

**Affiliations:** ^1^ Kibong’oto Infectious Diseases Hospital Kilimanjaro, Tanzania;; ^2^ Division of Infectious Diseases and International Health, University of Virginia, Charlottesville, USA;; ^3^ Kilimanjaro Christian Medical University College, Kilimanjaro, Tanzania;; ^4^ Ministry of Health, National Tuberculosis and Leprosy Programme, Dodoma,Tanzania;; ^5^ MKUTA National TB/HIV Patients Organization, Dar es Salam, Tanzania;; ^6^ The Nelson Mandela African Institution of Science and Technology (NM-AIST), Arusha, Tanzania;; ^7^ LHL International, Oslo, Norway.

**Keywords:** tuberculosis, Tanzania, lung health, pulmonary function test, 6 minute walk distance (6MWD), St Georges respiratory questionnaires, SGRQ

## Abstract

**BACKGROUND:**

Post-TB patients often experience persistent lung issues that impair exercise capacity and quality of life. Although pulmonary rehabilitation is known to be effective for chronic lung diseases, its role in post-TB lung disease remains underexplored in high TB-burden settings.

**METHOD:**

This prospective study (2021–2022) in Tanzania's Kilimanjaro region evaluated a 24-week, community-based pulmonary rehabilitation program led by TB survivors for adults with moderate-to-severe respiratory symptoms despite TB cure. The program included supervised exercise, breathing training, psychosocial support and smoking cessation. Outcomes measured at baseline, 12 weeks, and 24 weeks included spirometry, 6-minute walk distance (6MWD), St. George’s Respiratory Questionnaire (SGRQ), BMI, Generalized Anxiety Disorder-7 (GAD-7), and Patient Health Questionnaire-9 (PHQ-9).

**RESULTS:**

Among 121 participants (mean age 48±8.67 years, 89.2% male), significant improvements were observed in 6MWD (420 vs. 460 meters, p < 0.001) and SGRQ scores (34.63 to 12.99, p < 0.001). Smoking history predicted SGRQ improvement. Although no changes were seen in lung function or BMI, anxiety and depression symptoms improved in those with abnormal baseline scores.

**CONCLUSION:**

Community-based pulmonary rehabilitation improved symptomatic individuals' quality of life, physical capacity and mental health. Future research should refine intervention timing and evaluate long-term outcomes across diverse settings.

TB remains one of the most lethal infections worldwide.^1^ In the antibiotic era, most focus has been on improving cure rates, but many survivors continue to suffer a variety of respiratory challenges, profoundly impacting their quality of life (QoL) and posing a heightened risk of mortality.^2–5^ These challenges, collectively known as post-TB lung disease (PTLD), impact between 18–87% of people after TB is cured.^6,7^ The greatest burden of disability-adjusted life years lost among the 58 million people estimated to have PTLD in 2019, occurred in low-income countries (LICs).^4,8,9^ TB survivors have a three times higher risk of mortality compared to the general population,^10,11^ and PTLD-related physical and mental health issues lead to decreased economic capacity, decreased productivity and, in some cases, job loss.^6^

Factors increasing the risk of post-TB sequelae include delays in TB diagnosis, advanced lung damage at presentation, prolonged treatment due to interruptions or drug resistance, pre-existing undernutrition or inadequate nutrition during treatment, and inhalational exposures such as smoking or silica dust.^5,6,12,13^ Depending on the interplay among host and environmental predisposing factors, post-TB pathophysiology can lead to both reversible and irreversible alterations in pulmonary structure, function and immune signaling.^9–12^ Unlike some forms of chronic lung disease, PTLD may be marked by chronic inflammation and aberrant healing that culminates in a landscape of scarring, fibrosis and/or cavitation that can result in restrictive, obstructive or mixed patterns of lung injury.^14–20^ Symptom onset can be as late as six years after completion of TB treatment.^9^ Given the spectrum of PTLD, it is 1) critical to identify which subpopulations will benefit from treatment aimed at improving physical capacity, mental health and overall QoL, 2) determine which measurable factors in pre and post-TB predict the response to the interventions, and 3) test interventions in TB endemic settings with tools that are locally acceptable.

Pulmonary rehabilitation (PR) has been beneficial for certain chronic lung diseases, improving lung function, exercise endurance and QoL. It may also apply to people with PTLD.^21,22^ From 2021 onward, following more broad awareness of PTLD, expert groups have recommended early clinical assessment after TB treatment to stratify patients at risk for PTLD that may benefit from PR.^2,5,6,23^ Subsequent PR studies for PTLD have suggested benefits, but involve smaller numbers of participants with a range of functional decline pre-PR intervention or with PR carried out over short courses.^21,22,24,25^ Therefore, we sought to study the functional and QoL impact of a community-based and longer-term (24-week) PR intervention led by TB survivors in Tanzania among a prospective cohort with moderate-severe PTLD.

## METHODS

A 24-week prospective, non-controlled interventional study was conducted between October 2021 to December 2022 in the Kilimanjaro region of Tanzania to evaluate a community-based pulmonary rehabilitation (PR) program for adults with post-TB lung disease (PTLD) and severe symptoms. The study was coordinated through Kibong’oto Infectious Disease Hospital and organized by Mwitikio wa Kudhibiti Kifua Kikuu na Ukimwi Tanzania (MKUTA), a national organization combating TB and HIV. Aiming to enroll at least 600 participants, the study estimated that 60% would meet PTLD criteria, with 25% having moderate-severe symptoms, targeting 100 participants for the PR program. Screening included patients who had completed TB treatment within the last five years and had persistent respiratory symptoms. Patients with signs of recurrent TB, severe anemia, congestive heart failure, unstable angina, cardiac arrhythmias, or advanced cancer were excluded. Those eligible were further screened with chest X-rays and sputum tests such as sputum culture and Molecular bacteria load assay tests. Participants were categorized using the Modified Medical Research Council (MMRC) scale; those with moderate or severe symptoms (MMRC ≥ 2) were assigned to community-based, TB survivor-led PR, while those with less severe symptoms (MMRC < 2) received instructions for home-based PR. Antibiotic treatment for common bacterial pathogens did not preclude inclusion.However, after exclusions, no participants required a referral for specialized care

### Intervention

The PR bundle included supervised exercise sessions, education and psychosocial support.^26^ PR was done in community grounds (outdoors) and was delivered by trained members of a TB survivors group from MKUTA. The selected MKUTA members were community health workers with qualifications chosen to support this client-centered care. They underwent two weeks of theoretical and hands-on training to equip them with the necessary skills to deliver PR, followed by standardized assessment practices. Training was provided by physiotherapists, doctors, nurses, psychiatrists, and mental health counselors from Kibong’oto Infectious Diseases Hospital (KIDH) and Muhimbili National Hospital. During PR sessions, patients received financial stipends for transportation. PR was delivered 4 days per week in 90-minute sessions. The exercise included aerobic and resistance training, with a progressive increase in intensity and duration following patients’ tolerance (Supplementary Data Table S1). The education component discussed stress management, lung health, smoking cessation, energy conservation and breathing patterns training (box, pursed lip, rib stretching breathing, and diaphragmatic breathing).^27^ Psychosocial support with further evaluation was provided by counselors from the group of TB survivors for all patients who scored 8 or higher in their GAD-7 for anxiety^26^ or 5 or higher in PHQ9 for depression,^28,29^ during pre-PR (baseline) evaluation. A psychiatrist managed those with suicidal ideation or scores of ≥ 10 in GAD7 or PHQ9.

### Assessments

Body mass index (BMI) was measured, with <18.5 classified as malnourished. Mental health was assessed using GAD-7 for anxiety and PHQ-9 for depression, based on DSM-IV criteria. QoL was evaluated using the St. George’s Respiratory Questionnaire (SGRQ), designed for chronic lung disease, covering symptoms, activities and impacts. SGRQ scores range from 0 (best) to 100 (worst), with a total score ≥25 indicating an increased mortality risk from chronic obstructive pulmonary disease (COPD).^30,31^ The 6-minute walk test (6MWT) was conducted per American Thoracic Society (ATS) guidelines to measure functional capacity. Patients were instructed to walk as quickly as possible for 6 minutes, with the test discontinued if they experienced respiratory symptoms or were unable to continue.^2,22^ Pulmonary function testing was conducted using spirometry with the NDD Easy on PC spirometer, following Pan African Thoracic Society (PATS)-ATS-ERS 2019 guidelines. Decreases in FEV1/FVC and FEV1 indicate airway obstruction, while a decrease in FVC suggests restrictive lung abnormalities.^20^

### Analyses

The study assessed changes from baseline to week 24 in respiratory QoL (SGRQ), exercise tolerance (6MWT), nutritional status (BMI), mental health (GAD-7/PHQ-9), and pulmonary function (FEV1, FEV1/FVC). Differences were analyzed using Mann-Whitney U and Kruskal-Wallis tests. Secondary analyses explored predictors of outcomes using logistic regression, including baseline pulmonary function's impact on SGRQ scores. Patients’ acceptance of the PR program was evaluated using a Likert scale. Data analysis was conducted using IBM SPSS Statistics version 29. Baseline factors influencing PR response and SGRQ component scores were also examined.

This study complied with the protocol and regulatory requirements approved by the Institutional Review Board (IRB) KNCHREC001. Eligible participants completed written informed consent.

## RESULTS

**FIGURE 1.**
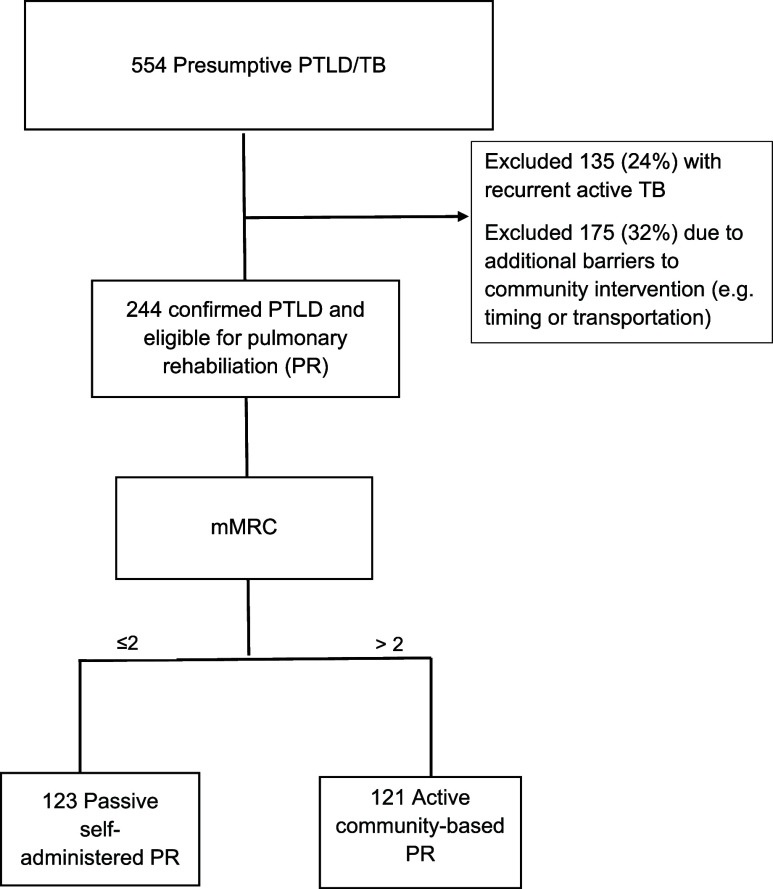
Screening and enrollment of participants. PTLD = post-TB lung disease; PR = Pulmonary rehabilitation; mMRC = Modified Medical Research Council dyspnea scale.

Between October 2021 to December 2022, 625 participants (Figure 1) with a history of TB and symptoms were screened, with 523 (84%) identified through community outreach. Of these, 234 were diagnosed with PTLD and met the criteria for PR. Among them, 121 participants with mMRC scores ≥2 underwent a TB-survivor-led community-based PR program, while those with mild symptoms received home-based PR instructions. The mean age was 48±8.67 years, 89% were male and the most common occupation was mining. Baseline spirometry showed mixed patterns in 38%, normal in 25%, obstructive in 11%, restrictive in 9%, and unclassifiable in 17% (Table 1).

**TABLE 1.** Baseline demographics of participants (N =121) during the study.

Demographics	Number (%)
Age mean (SD)	48 (8.67)
Gender: Male n (%)	105 (89.2)
Marital status n (%)	
Married	84 (70)
Single	36 (33.6)
BMI (kg/*mm*^22^) mean (SD) <18.5	36 (33.6)
≥18.5	84 (78.5)
*Education n (%) level*	
< secondary education	116 (96.7)
≥ secondary education	4 (3.3)
*Occupation n (%)*	
Mining	54 (44.6)
Farming	32 (26.4)
*Smoking History n (%)*	69 (64.3)
*History of firewood/charcoal exposure n (SD)*	71 (59.7)
*Comorbidities mean (SD)*	
Diabetes	6 (5)
HIV	14 (11.7)
*Time since TB cure, median years (IQR)*	4 (2-4)
*Spirometry pattern n (%)*	
Normal	30 (25)
Obstructive	13 (11)
Mixed	46 (37.5)
Restrictive	11 (9.2)
Poor quality	21(17.4)

SD = standard deviation; IQR = interquartile range.

Before starting PR, 39 (32%) participants were treated for non-TB pneumonia, with pathogens including *Klebsiella pneumoniae* (33%), *Pseudomonas aeruginosa* (26%), and methicillin-sensitive *Staphylococcus aureus* (21%), *Streptococcus pneumonia (*5%), *Moraxella catarrhalis* (3%) and *Acinetobacter baumanii* (3%). All 121 participants completed baseline, 12-week, and 24-week assessments and over 90% of PR sessions. Supplemental Table 2 describes post-PR evaluations showing high satisfaction, with participants agreeing or strongly agreeing on the program's efficacy and structure.

### Primary outcome

From baseline to week 24, there was a significant improvement in respiratory QoL, with a mean total SGRQ score of 34.63±17.03 at baseline and 12.99±9.67 at week 24. Walking distance increased significantly by 40 meters in 6 minutes, from 420±87.30 meters at baseline to 460±61.96 meters at week 24 (Table 2). The mean component scores of the SGRQ at baseline, week 12, and week 24 are shown in Supplementary Data Figure S1**.** Mean BMI, GAD-7, PHQ-9, and potentially reversible patterns of pulmonary function (FEV1 and FEV1/FVC) did not significantly improve from baseline to week 24. When excluding those with no reported anxiety or depression symptoms at baseline (GAD-7 and PHQ-9 scores of 0), n= 68 remained, and of those a significant improvement in both scores of mental health were observed (Supplementary Data Figure S2). Mean ± (SD) GAD-7 improved from baseline of 9.97 (5.70) to week 12 of 7.11 (5.014), and week 24 of 6.39 (5.13) p = 0.001, and mean ± (SD) of PHQ-9 improved from baseline of 8.71 (6.31) to week 12 of 5.6 (5.40), to week 24 of 4.80 (4.35) p = 0.039.

**TABLE 2.** Primary outcomes following pulmonary rehabilitation (N = 121).

Outcomes	Baseline	Week 12	Week 24	p=value
*Spirometry pattern*				
% predicted FEV1 median [IQR]	67.00 [49–87.25]		69.50 [51.25–91.75]	0.288
% predicted FVC median [IQR]	74.00 [59.75–93.25]		77.00 [62.25–98.75]	0.189
Lower Limit Normal FEV1/FVC Median [IQR]	69.60 [68.20–70.70]		69.40 [68.20–70.70]	0.400
*Exercise capacity (m)*				
6MWD mean (SD)	420 (87.30)	455 (66.80)	460 (61.96)	<0.001
*Nutritional assessment*				
BMI Median [IQR]	20.6 [18.1-24.30]	21.0 [18.7-24.18]	21.2 [19.00–23.90]	0.578
*Respiratory quality of life measure mean (SD)*				
SGRQS	45.68 (16.56)	26.37 (14.64)	21.10 (13.31)	<0.001
SRQA	46.98 (22.70)	26.13 (15.12)	17.44 (13.47)	<0.001
SGRQI	22.99 (14.99)	10.47 (9.47)	6.06 (5.63)	<0.001
SGRQT	34.63 (17.03)	19.14 (11.04)	12.99 (9.67)	<0.001
*Mental health*				
PHQ-9 mean (SD)	2.10 (4.575)	2.18 (4.031)	2.20 (3.504)	0.136
GAD-7 mean (SD)	6.75 (6.37)	8.19 (3.560)	7.88 (3.45)	0.118

Displays the changes in outcome variables of patients in pre-(baseline) and post- PR (week 12 and week 24). FEV1 (forced expiratory volume in one second)/FVC (forced vital capacity) were recorded at the beginning of the study. 6MWD = 6-minute walk distance; body mass index = BMI; HRQoL = health-related quality of life, measured with St George Respiratory score (SGRQ). SGRQ-S represents the symptoms score, SGRQ-I represents the impact score, SGRQ-A represents the activity score, and SGRQ-T represents the total score in St. George Respiratory Questionnaires.Interquartile range [IQR]. SD = Standard deviation.

### Secondary and exploratory outcomes

Regression analyses identified predictors of improvement in SGRQ and 6MWD following PR. Baseline demographics, including gender, occupation, firewood exposure, and comorbidities (diabetes, HIV, mental illness), were considered. Smoking history predicted SGRQ improvement but not 6MWD. Among 69 smokers, 66 (94%) improved in SGRQ total by week 24. In contrast, farming predicted no response to PR, with no improvement in SGRQ or 6MWD. Sub-analysis attributed this to baseline SGRQ scores, which were higher in smokers (34) than in farmers (24). Baseline pulmonary function patterns were weakly associated with lower SGRQ total and component scores (activity, symptoms, impact), suggesting better respiratory QoL with higher lung function (Table 3).

**TABLE 3.** Baseline demographic associations with quality of life and six-minute walk distance response to pulmonary rehabilitation.

Predictors	SGRQ-Odds ratio [95% CI]	p- value ϒ	SGRT- Odds ratio [95% CI]	p- value ¥	6MWD [95% CI]	p- value ϒ	6MWD [95% CI]	p- value ¥
Male	6.35	.010	0.539	0.577	0.745	0.715	0.448	0.417
	[1.558, 25.880]		[0.062, 4.717]		[0.153, 3.61]		[0.064, 3.118]	
History of	4.71	0.026	8.245	0.022	1.622	0.299	1.943	0.225
Smoking	[1.21,18.42]		[1.362, 49.914]		[0.651, 4.044]		[0.665, 5.674]	
History of	0.831	0.778	0.772	0.785	1.622	0.309	1.676	0.339
firewood exposure	[0.229, 3.011]		[0.120, 4.95]		[0.639, 4.113]		[0.581, 4.832]	
Mining	*	*	*	*	2.149	0.124	2.238	0.217
					[0.812, 5.687]		[0.624, 8.034]	
Farming	0.025	<0.001	0.045	0.008	0.381	0.047	0.451	0.203
	[0.003, 0.208]		[0.004, 0.451]		[0.147, 0.988]		[0.132, 1.537]	
HIV	0.563	0.493	0.606	0.668	3.445	0.245	3.268	0.300
	[0.109, 2.911]		[0.62, 5.962]		[0.427, 27.79]		[0.348, 30.695]	
Diabetes	0.481	0.522	2.209	0.588	0.452	0.377	0.464	0.447
	[0.51, 4.529]		[0.146, 29.832]		[0.078, 2.631]		[0.064, 3.360]	

Univariable and multivariable binomial logistic regression of predictors of improvement in St. Georges respiratory questionnaire total (SGRQT) and 6-minute walk distance (6MWD) PR = pulmonary rehabilitation expressed as odd ratios; CI = confidence interval;

ϒ = univariable;

¥ := multivariable;

* = Noncalculable.

## DISCUSSION

PR is critical for mitigating post-TB pulmonary sequelae, encompassing interventions such as exercise, breathing techniques, smoking cessation, psychosocial counseling, and nutritional recommendations.^32^ Identifying which PR components drive specific health benefits and in which subpopulations can be challenging, particularly in PTLD, where lung injury patterns vary (obstructive, restrictive, or mixed) and disease severity ranges from minimal to debilitating. This study focused on community-dwelling adults with moderate to severe PTLD symptoms in a high TB burden region and demonstrated that a 24-week community-based, TB survivor-led PR program significantly improved respiratory QoL and walking distance. The high adherence and rigorous outcome measurement at baseline, 12 weeks, and 24 weeks bolster our confidence in these associations. As the median time from completion of prior TB treatment to the PR intervention was 4 years, these improvements were unlikely to be from natural history alone.

The proportional improvement in QoL exceeded that reported in other PTLD studies, likely due to the extended PR duration which allowed more time for behavioral changes and mood improvements. The comprehensive PR approach, involving physical and psychosocial modalities administered by TB survivor peers, also effectively supported smoking cessation and normalized healthy behaviors across the population. All SGRQ components (Supplementary Data Figure 1a-c) improved significantly, with PR being most impactful for those with more advanced PTLD. Nevertheless, the directionality of improvement in QoL and walking distance measured were consistent with other smaller PR studies in PTLD.^21,22,24,25,33^ Singh et al. administered PR in 29 participants with PTLD in India, over 8 weeks of 3x-weekly sessions and found a mean increase in 6MWD of 38 meters, comparable to our findings of a 40-meter increase after 24 weeks.^22^ Similarly, Jones et al. administered PR to 34 patients with PTLD in Uganda, and showed improvements in the incremental shuttle walking test and QoL as measured by the Clinical COPD Questionnaire following six weeks of 2x-weekly sessions.^21^ Additionally, Visca et al.'s retrospective study of 43 patients with PTLD in Italy found that 34 patients with impaired respiratory patterns saw a mean increase in 6MWD from 371 to 406 meters after three weeks of 5x-weekly PR.^24^

**FIGURE 2.**
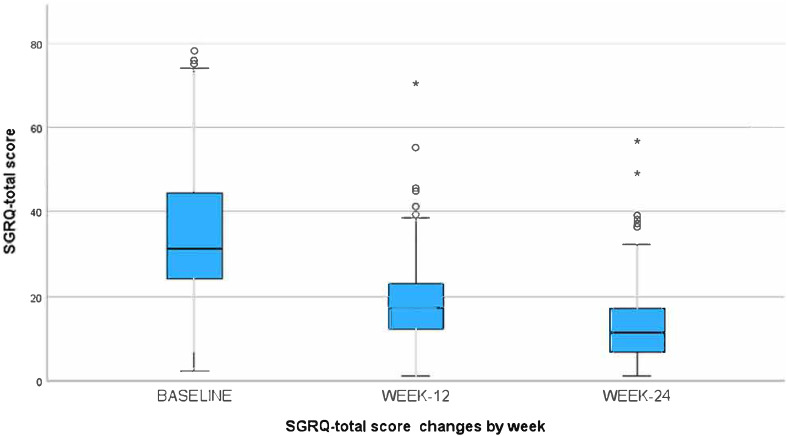
St. George’s Respiratory Questionnaire (SGRQ), SGRQ-total score at baseline, week 12, and week 24, showing improved quality of life following pulmonary rehabilitation.

**FIGURE 3.**
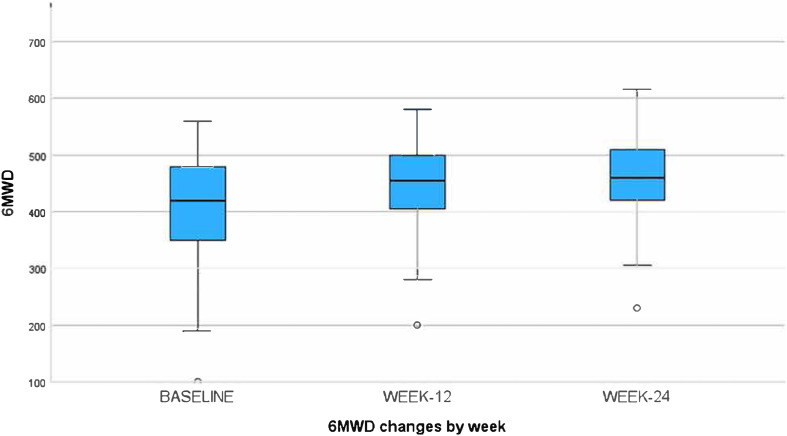
Six-minute walk distance (6MWD) improvements were observed from baseline through weeks 12 and 24 of rehabilitation.

We also observed that certain subgroups with higher baseline SGRQ total scores did not achieve significant improvement with PR. Despite improvements in the SGRQ total score and 6MWD (Table 2 and Figures 2 & 3), pulmonary function did not improve after 24 weeks (FVC, FEV1, and FEV1/FVC). This aligns with a recent systematic review and meta-analysis that indicated that PR in post-TB treatment did not improve lung function^.32^ Although our study shows PR improves QoL and exercise capacity, further research should explore the ideal timing, during or after TB treatment, to assess its impact on reversible lung deficits, especially as global efforts emphasize the need to integrate long-term outcome into TB care and trials, as underscored by a recent international post-TB symposium.^33^

Unexpectedly, our study did not observe improvements in mental health markers (Table 2) across the total population as observed in other PR settings in PTLD.^34,35^ When restricting analysis only to those reporting anxiety or depression at baseline, scores did significantly improve for these subpopulations (Supplementary Data Figure S2a-b). While other studies used more generalized measurements of mental health tested within other QoL metrics, we choose to employ the more disease-specific scoring systems for anxiety and depression with GAD-7 and PHQ-9, respectively. Although we may have more accurately quantified trends in anxiety and depression symptoms that prior studies overestimated with other tools, we suspect that our findings were primarily influenced by a sociocultural reticence to rate mental health at baseline, which lessened over the weeks of PR as participants worked with peers in an accepting environment as suggested by the favorable programmatic evaluation (Supplementary Data Table S2).

We did not anticipate significant BMI improvements, as the population included individuals up to five years post-TB treatment, removed from the undernourishment of TB illness. BMI may remain unchanged despite improved dietary diversity, caloric intake, exercise, or muscle mass gain. Detailed assessments, such as mean upper arm circumference, DEXA scans, or bioelectric impedance assays, could provide better estimates of total body water, fat mass, and body cell mass, clarifying whether dietary changes with PR lead to sustained nutritional benefits.^36^

Despite the strengths of our study being based in a high-TB-burden region, the study had several limitations due to its noncontrolled design. A randomized design was excluded over behavioral spillover concerns. Outcomes were not compared with 113 participants (mMRC <2) receiving home-based instructions due to differing of PTLD severity. Scaling PR across Tanzania, where MKUTA TB survivor groups are active, could benefit from a cluster randomized design focusing on SGRQ and 6MWD. A subgroup had sputum cultures positive for pneumonia-related pathogens, complicating PR effect isolation as some received concurrent antibiotics. However, continued improvement from week 12 to 24 suggests lasting PR benefits beyond antibiotics, reinforcing its potential in PTLD management.

In conclusion, our study in Tanzania showed that a 24-week community-based, peer-led PR program improved QoL, walking distance and mood in PTLD patients with anxiety or depression. Further trials should refine optimal PR timing and its impact on lung function. Programs should consider PR for moderate to severe PTLD cases.
